# Air Pollution Exposure during Pregnancy and Childhood Autistic Traits in Four European Population-Based Cohort Studies: The ESCAPE Project

**DOI:** 10.1289/ehp.1408483

**Published:** 2015-06-12

**Authors:** Mònica Guxens, Akhgar Ghassabian, Tong Gong, Raquel Garcia-Esteban, Daniela Porta, Lise Giorgis-Allemand, Catarina Almqvist, Aritz Aranbarri, Rob Beelen, Chiara Badaloni, Giulia Cesaroni, Audrey de Nazelle, Marisa Estarlich, Francesco Forastiere, Joan Forns, Ulrike Gehring, Jesús Ibarluzea, Vincent W.V. Jaddoe, Michal Korek, Paul Lichtenstein, Mark J. Nieuwenhuijsen, Marisa Rebagliato, Rémy Slama, Henning Tiemeier, Frank C. Verhulst, Heather E. Volk, Göran Pershagen, Bert Brunekreef, Jordi Sunyer

**Affiliations:** 1 Center for Research in Environmental Epidemiology (CREAL), Barcelona, Spain; 2 Pompeu Fabra University, Barcelona, Spain; 3 Spanish Consortium for Research on Epidemiology and Public Health (CIBERESP), Instituto de Salud Carlos III, Madrid, Spain; 4 Department of Child and Adolescent Psychiatry/Psychology, Erasmus University Medical Centre–Sophia Children’s Hospital, Rotterdam, the Netherlands; 5 The Generation R Study, Erasmus University Medical Centre, Rotterdam, the Netherlands; 6 Department of Medical Epidemiology and Biostatistics, Karolinska Institutet, Stockholm, Sweden; 7 Department of Epidemiology Lazio Regional Health Service, Rome Italy; 8 Team of Environmental Epidemiology Applied to Reproduction and Respiratory Health, Inserm (National Institute of Health and Medical Research) (U823), Grenoble, France; 9 University Grenoble-Alpes, Institut Albert Bonniot, Grenoble, France; 10 Psychobiology area, Department of Basic Psychological Processes and Their Development, Faculty of Psychology, University of The Basque Country (UPV/EHU), Donostia-San Sebastian, Spain; 11 Health Research Institute BIODONOSTIA, Donostia-San Sebastian, Spain; 12 Institute for Risk Assessment Sciences, Utrecht University, Utrecht, the Netherlands; 13 Centre for Environmental Policy, Imperial College London, London, UK; 14 Foundation for the Promotion of Health and Biomedical Research in the Valencian Region, University of Valencia–University Jaume I Joint Research Unit of Epidemiology and Environmental Health, Valencia, Spain; 15 Department of Genes and Environment, Division of Epidemiology, Norwegian Institute of Public Health, Oslo, Norway; 16 Sub-Directorate of Public Health of Gipukzoa, Department of Health, Government of Basque Country, San Sebastian, Spain; 17 Department of Pediatrics, Erasmus University Medical Centre–Sophia Children’s Hospital, Rotterdam, the Netherlands; 18 Department of Epidemiology, Erasmus University Medical Centre, Rotterdam, the Netherlands; 19 Institute of Environmental Medicine, Karolinska Institutet, Stockholm, Sweden; 20 Department of Medicine, University Jaume I, Castelló de la Plana, Spain; 21 Department of Psychiatry, Erasmus University Medical Centre, Rotterdam, the Netherlands; 22 Department of Preventive Medicine, and; 23 Department of Pediatrics, Keck School of Medicine, University of Southern California, Los Angeles, Los Angeles, California, USA; 24 Children’s Hospital Los Angeles, Los Angeles, California, USA; 25 Julius Center for Health Sciences and Primary Care, University Medical Center Utrecht, the Netherlands; 26 Hospital del Mar Research Institute, Barcelona, Spain

## Abstract

**Background:**

Prenatal exposure to air pollutants has been suggested as a possible etiologic factor for the occurrence of autism spectrum disorder.

**Objectives:**

We aimed to assess whether prenatal air pollution exposure is associated with childhood autistic traits in the general population.

**Methods:**

Ours was a collaborative study of four European population-based birth/child cohorts—CATSS (Sweden), Generation R (the Netherlands), GASPII (Italy), and INMA (Spain). Nitrogen oxides (NO_2_, NO_x_) and particulate matter (PM) with diameters of ≤ 2.5 μm (PM_2.5_), ≤ 10 μm (PM_10_), and between 2.5 and 10 μm (PM_coarse_), and PM_2.5_ absorbance were estimated for birth addresses by land-use regression models based on monitoring campaigns performed between 2008 and 2011. Levels were extrapolated back in time to exact pregnancy periods. We quantitatively assessed autistic traits when the child was between 4 and 10 years of age. Children were classified with autistic traits within the borderline/clinical range and within the clinical range using validated cut-offs. Adjusted cohort-specific effect estimates were combined using random-effects meta-analysis.

**Results:**

A total of 8,079 children were included. Prenatal air pollution exposure was not associated with autistic traits within the borderline/clinical range (odds ratio = 0.94; 95% CI: 0.81, 1.10 per each 10-μg/m^3^ increase in NO_2_ pregnancy levels). Similar results were observed in the different cohorts, for the other pollutants, and in assessments of children with autistic traits within the clinical range or children with autistic traits as a quantitative score.

**Conclusions:**

Prenatal exposure to NO_2_ and PM was not associated with autistic traits in children from 4 to 10 years of age in four European population-based birth/child cohort studies.

**Citation:**

Guxens M, Ghassabian A, Gong T, Garcia-Esteban R, Porta D, Giorgis-Allemand L, Almqvist C, Aranbarri A, Beelen R, Badaloni C, Cesaroni G, de Nazelle A, Estarlich M, Forastiere F, Forns J, Gehring U, Ibarluzea J, Jaddoe VW, Korek M, Lichtenstein P, Nieuwenhuijsen MJ, Rebagliato M, Slama R, Tiemeier H, Verhulst FC, Volk HE, Pershagen G, Brunekreef B, Sunyer J. 2016. Air pollution exposure during pregnancy and childhood autistic traits in four European population-based cohort studies: the ESCAPE Project. Environ Health Perspect 124:133–140; http://dx.doi.org/10.1289/ehp.1408483

## Introduction

Autism spectrum disorders (ASD) are lifelong developmental disabilities characterized by social interaction impairment, communication deficits, and repetitive behaviors (van Engeland and Buitelaar 2008). The prevalence of ASD has increased in the past 20 years, reaching 1 in 86 children in Europe in 2007 (Posada et al. 2007). Despite advances in genetic research, the causes of ASD remain unclear (Betancur 2011). A possible etiologic role for environmental factors has been suggested, particularly during pregnancy (Dietert et al. 2011).

Two recent case–control studies in California showed that ASD in children 2–6 years of age was associated with prenatal exposure to traffic-related air pollutants (Becerra et al. 2013; Volk et al. 2011, 2013), but the results of a twin study from Sweden did not confirm that finding (Gong et al. 2014). Another case–control study among children of Nurses’ Health Study II participants reported an association between prenatal exposure to PM_2.5_ (particulate matter with aerodynamic diameter ≤ 2.5 μm) (Raz et al. 2015) and other air pollutants such as diesel or metals at birth (Roberts et al. 2013) and ASD. Two case–control studies were also carried out in the United States, one showing a significant association of ASD with higher ambient air concentrations of metals at birth (Windham et al. 2006), and another showing null associations between several pollutants at birth and ASD after adjusting for confounders (Kalkbrenner et al. 2010). Brain toxicity of urban air pollutants during development is well documented in animals, and possible biological pathways have been suggested (Block et al. 2012).

Autistic traits are defined as subclinical deficits in socialization, communication, and repetitive behaviors that do not meet formal criteria for an ASD diagnosis (Constantino and Todd 2003). It has been shown that known genetic and environmental influences are consistent across the range of impairment of the continuous autistic trait, indicating an etiologic overlap between very extreme scores, mild impairment, and subthreshold autism-like behavior (Robinson et al. 2011). To date, no study has examined the association of air pollution with the presence of autistic quantitative traits in the general population. In this study we aimed to assess whether prenatal air pollution exposure including nitrogen dioxide (NO_2_) and PM is associated with autistic traits in childhood in four European population-based birth/child cohort studies.

## Methods

*Population and study design*. This study was part of the European Study of Cohorts for Air Pollution Effects (ESCAPE), in which the association between exposure to outdoor air pollution and health is being investigated within prospective cohort studies (http://www.escapeproject.eu). We included three European population-based birth cohorts: Generation R (the Netherlands) (Jaddoe et al. 2012), GASPII (Gene and Environment: Prospective Study on Infancy in Italy) (Porta et al. 2006), and INMA (INfancia y Medio Ambiente; Childhood and Environment; Spain, including three subcohorts) (Guxens et al. 2012), and a European longitudinal child and adolescent twin study: CATSS (Child and Adolescent Twin Study in Sweden) (Anckarsäter et al. 2011) (Table 1). Mother–child pairs were recruited from 1992 through 2008. A total of 8,079 children with available data on exposures, outcome, and potential confounders were included (62.2% of the children recruited at baseline). Informed consent was obtained from all participants in each cohort and ethical approval was obtained from the local authorized institutional review boards.

**Table 1:** Description of the participating birth cohort studies.

Cohort study	Setting		Air pollution		Autistic traits					
	Location	Pregnancy period	Pollutants	% of birth addresses in urban areas	Test	Age (years)	Evaluator	*n*^*a*^	*n* (%) within borderline or clinical range	*n* (%) within clinical range only
CATSS	Stockolm (Sweden)	1992–2000	NO_2_, NO_x_, PM, traffic intensity, traffic load	45.7	ASD module (A-TAC)	10	Parents	2,437	78 (3.2)	27 (1.1)
Generation R	Rotterdam (the Netherlands)	2001–2005	NO_2_, NO_x_, PM, traffic intensity, traffic load	100.0	PDP subscale (CBCL½–5)	6	Parents	3,955	336 (8.5)	143 (3.6)
					Adapted 18‑item version of SRS	6	Parents	3,231	NA^*b*^	NA^*b*^
GASPII	Rome (Italy)	2003–2004	NO_2_, NO_x_, PM, traffic intensity, traffic load	100.0	PDP subscale (CBCL½–5)	4	Parents	514	63 (12.3)	15 (2.9)
INMA	Gipuzkoa (Spain)	2006–2008	NO_2_, NO_x_	89.1	CAST	4	Psychologist	357	17 (4.8)	3 (0.8)
	Sabadell (Spain)	2004–2006	NO_2_, NO_x_, PM, traffic load	100.0	CAST	4	Psychologist	295	10 (3.4)	2 (0.7)
	Valencia (Spain)	2004–2005	NO_2_, NO_x_, traffic load	92.7	CAST	5	Psychologist	521	37 (7.1)	10 (1.9)
Abbreviations: ASD, autism spectrum disorder; A-TAC, Autism–Tics, Attention Deficit and Hyperactivity Disorders, and Other Comorbidities Inventory; CBCL, Child Behavior Checklist; CAST, Childhood Autism Spectrum Test; NO_2_, nitrogen dioxide; NO_x_, nitrogen oxides; PDP, pervasive developmental problems; PM, PM ≤ 10 μm (PM_10_), PM ≤ 2.5 μm (PM_2.5_), PM between 2.5 and 10 μm (PM_coarse_), PM_2.5_absorbance (reflectance of PM_2.5_ filters); SRS, Social Responsiveness Scale; Traffic intensity, traffic intensity on the nearest road; Traffic load, total traffic load (intensity × length) on all major roads within 100-m buffer. ^***a***^Number of children with air pollution, autistic traits, and potential confounders variables available. ^***b***^NA, not applicable because the cut-off points for autistic traits within the borderline/clinical range and within the clinical range have not been defined for the 18-item version of the SRS; score evaluated only as a continuous quantitative outcome.										

*Air pollution exposure*. Air pollution concentrations at the participants’ birth home addresses were estimated for the whole pregnancy period of each woman by land-use regression models following a standardized procedure described elsewhere (Beelen et al. 2013; Eeftens et al. 2012a) (see Supplemental Material, “Methods S1. Description of the air pollution assessment”). Briefly, air pollution monitoring campaigns were performed in the study areas between October 2008 and January 2011. In all areas, three 2-week measurements of NO_2_ and nitrogen oxides (NO_x_) were performed within 1 year (Cyrys et al. 2013). In all cohorts except in the Spanish cohorts of Valencia and Gipuzkoa, simultaneous measurements of PM_10_ (PM with aerodynamic diameter ≤ 10 μm), PM_2.5_, PM with aerodynamic diameters between 2.5 and 10 μm (PM_coarse_), and PM_2.5_ absorbance (determined as the reflectance of PM_2.5_ filters) were performed (Eeftens et al. 2012b) (Table 1). We developed land-use regression models for each pollutant metric using all measurement sites and used them to estimate annual average air pollution concentration at the participants’ birth home addresses. We used a back-extrapolation procedure to estimate pregnancy-average concentrations from annual average concentration using routine background monitoring network sites (Pedersen et al. 2013). Traffic intensity on the nearest road and total traffic load (intensity × length) on all major roads within a 100-m buffer were available for some cohorts.

*Autistic traits*. Autistic traits were assessed in children using the Autism Spectrum Disorder module of the Autism—Tics, Attention Deficit and Hyperactivity Disorders, and Other Comorbidities (A-TAC) inventory (Anckarsäter et al. 2011) in the Swedish cohort at age 9 or 12 years; the Pervasive Developmental Problems (PDP) subscale of the Child Behavior Checklist for Toddlers (CBCL1½–5) (Achenbach and Rescorla 2000) in the Dutch cohort at age 6 years and in the Italian cohort at age 4 years; an adapted 18-item version of the Social Responsiveness Scale (SRS) (Constantino and Gruber 2005; Román et al. 2013) in the Dutch cohort at age 6 years; and the Childhood Autism Spectrum Test (CAST) (Baron-Cohen et al. 2009) in the Spanish cohorts at age 4–5 years (Table 1; see also Supplemental Material, “Methods S2. Description of the autistic traits assessment” and Table S1). The A-TAC, the CBCL1½–5, and the adapted 18-item SRS were parent-reported questionnaires, whereas the CAST was a questionnaire administered to the parents by a psychologist. For all tests, higher scores indicated more autistic traits. We considered all scores as quantitative traits. We also used validated cut-offs to yield proxies for autistic traits within the borderline/clinical (borderline or clinical) range and within the clinical range only, specific for each test (Larson et al. 2010; Tick et al. 2007; Williams et al. 2005), except for the adapted 18-item SRS, for which these cut-offs are not defined. Validation studies reported high sensitivity (0.85–0.99) for borderline/clinical cut-offs and high specificity (0.95–0.97) for clinical cut-offs (see Supplemental Material, “Methods S2. Description of the autistic traits assessment” and Table S1).

*Potential confounding variables*. Potential confounding variables were defined *a priori* as similarly as possible across cohorts, given available information. Maternal characteristics were collected by questionnaires during pregnancy or at birth: age at delivery, educational level (≤ 9, 10–12, ≥ 12 years in the Swedish cohort; ≤ 11, 12–15, ≥ 16 years in the Spanish cohort; primary, secondary, or ≥ university in the Dutch and Italian cohorts), country of birth, prenatal smoking, and parity. Maternal height and prepregnancy weight were measured or self-reported in the first trimester of pregnancy or at birth. Prepregnancy body mass index (kilograms per meter squared) was calculated. Child’s sex and date of birth were obtained from hospital or national registries. We also collected child’s age at autistic trait assessment and information on the evaluator (parents, psychologist) of the autistic traits. Urbanicity at child’s birth address (urban, rural) was defined (urban classification: municipalities with > 40 inhabitants per hectare in the Swedish cohort; municipalities with > 2,000 inhabitants in the Dutch, Italian, and Spanish cohorts). Mothers reported on changes in residence (since birth until autistic trait assessment) through questionnaires.

*Statistical analyses*. All analyses were performed following a consensus protocol. We used logistic regression models to assess the association between air pollution exposure and autistic traits within the borderline/clinical and within the clinical range. For both analyses we considered children with scores below the borderline cut-off as controls. Because few children were classified as having autistic traits within the clinical range in the Spanish cohorts of Gipuzkoa and Sabadell, we did not include them in that analysis. We used negative binomial regression models to assess the association between air pollution exposure and autistic traits as a quantitative score. Models for the Swedish cohort include a random intercept to take into account that children were clustered in twin pairs.

First, models were adjusted for child’s age and sex (minimally adjusted models). When child’s age did not have a linear relationship with the autistic traits scales, we used the best transformation of the age found using fractional polynomials. Second, models were additionally adjusted for all covariates described above (fully adjusted models). Generalized additive models were used to assess the linearity of the relationship between each air pollutant and autistic trait scales by graphical examination and deviance comparison. Linear function provided a good fit in all cases. Spatial clustering of observations was explored by adding random cohort-level intercepts (Swedish cohort: small administrative units; Dutch cohort: neighborhood; Italian and Spanish cohorts: census area) to fully-adjusted models without the air pollution data. The inclusion of the spatial clustering component had a negligible impact on the Akaike Information Criterion. We used a two-stage approach to estimate the associations of air pollution exposure on autistic traits in children. First, associations were analyzed separately for each cohort. Second, cohort-specific effect estimates from the logistic regression models were combined using random-effects meta-analysis. We assessed heterogeneity in the estimates using the *Q* test and the *I^2^* statistic. Because quantitative scales of the autistic trait tests used in different cohorts did not share a common metric, meta-analyses of cohort-specific effect estimates on the autistic traits as continuous variables were not possible.

We performed several sensitivity analyses: *a*) meta-analyses leaving out one cohort at a time to determine the influence of a particular cohort, *b*) meta-analyses including the cohorts with information on both NO_2_ and PM (89% of the children), *c*) meta-analyses using the 90th percentile as a cut-off regardless of the scale because borderline/clinical and clinical cut-offs were specific to the scale used in each cohort, *d*) meta-analyses stratified by type of evaluator of the autistic traits (psychologist, parents), *e*) meta-analyses assessing the non–back-extrapolated air pollution variables, *f*) meta-analyses including children who had a stable residence from birth until the autistic traits assessment, *g*) meta-analyses restricted to children of highly educated mothers and meta-analysis restricted to children of mothers who did not smoke during pregnancy in order to assess potential modifications of the air pollution effects by these factors, and *h*) meta-analyses stratified by child’s sex because some studies found different association in boys and girls. Power sample calculation can be found in the Supplemental Material, Table S2. Statistical tests of hypotheses were two-tailed with significance set at *p* < 0.05. Statistical analyses were conducted using STATA (version 12.1; StataCorp, College Station, TX, USA).

## Results

Between 3.2% and 12.3% of children were classified as having autistic traits within the borderline/clinical range, and between 0.7% and 3.6% were classified as having autistic traits within the clinical range (Table 1). Children defined as having autistic traits within the borderline/clinical range or within the clinical range showed consistent associations with the assessed child and maternal characteristics across all cohorts (Table 2). Children who had autistic traits within the borderline/clinical range and within the clinical range were mostly boys and had a higher proportion of mothers with low educational level and mothers who smoked during pregnancy compared with children without autistic traits (Table 2).

**Table 2:** Distribution of the child and maternal characteristics.

Cohort	*n*	Child’s sex (% female)	Maternal educational level (%)			Maternal country of birth (% foreign)	Maternal age at delivery [years (mean ± SD)]	Maternal prepregnancy body mass index [kg/m^2^ (mean ± SD)]	Maternal height [cm (mean ± SD)]	Prenatal maternal smoking (% smokers)	Parity (% nulliparous)
			Low	Medium	High						
All population
CATSS, Sweden	2,437	49.1	10.5	48.6	40.9	13.0	31.6 ± 4.6	23.5 ± 3.6	167.5 ± 6.2	14.3	23.6
Generation R, the Netherlands	3,955	50.4	6.1	38.1	55.8	37.2	31.6 ± 4.6	24.3 ± 4.1	168.7 ± 7.3	12.7	59.4
GASPII, Italy	514	49.0	10.3	51.2	38.5	3.9	33.8 ± 4.2	22.2 ± 3.4	164.6 ± 5.9	10.7	57.0
INMA, Spain-Gipuzkoa	357	50.1	11.2	36.4	52.4	2.5	32.8 ± 3.4	22.9 ± 3.5	163.6 ± 6.0	23.2	55.5
INMA, Spain-Sabadell	295	49.8	26.8	35.6	37.6	8.1	31.7 ± 4.1	23.5 ± 4.5	162.4 ± 5.8	27.1	60.3
INMA, Spain-Valencia	521	48.0	27.8	43.8	28.4	8.1	31.8 ± 4.2	23.8 ± 4.4	162.2 ± 6.3	37.6	54.1
CATSS, Sweden
Children without autistic traits	2,359	50.0	10.2	48.2	41.6	13.1	31.7 ± 4.6	23.5 ± 3.6	167.5 ± 6.2	14.0	23.3
Children within the borderline or clinical range	78	23.1	21.8	59.0	19.2	7.7	30.4 ± 4.6	24.6 ± 5.1	167.0 ± 6.7	23.1	33.3
Children within the clinical range only	27	18.5	18.5	66.7	14.8	11.1	30.3 ± 4.2	27.2 ± 6.8	165.3 ± 7.1	25.9	29.6
Generation R, the Netherlands
Children without autistic traits	3,619	51.8	5.6	37.7	56.6	36.3	31.7 ± 4.5	24.3 ± 4.1	168.8 ± 7.2	12.1	58.8
Children within the borderline or clinical range	336	36.0	11.0	42.0	47.0	47.9	30.5 ± 5.0	24.3 ± 4.6	167.6 ± 7.6	18.5	66.7
Children within the clinical range only	143	26.6	12.6	42.0	45.5	51.7	30.1 ± 5.2	24.5 ± 5.1	166.5 ± 7.8	19.6	69.2
GASPII, Italy
Children without autistic traits	451	50.1	9.1	51.2	39.7	4.4	33.8 ± 4.2	22.1 ± 3.4	164.6 ± 5.9	11.1	55.2
Children within the borderline or clinical range	63	41.3	19.0	50.8	30.2	0.0	33.7 ± 4.2	23.0 ± 3.6	164.5 ± 5.9	7.9	69.8
Children within the clinical range only	15	60.0	20.0	60.0	20.0	0.0	34.6 ± 3.4	22.9 ± 3.2	165.1 ± 6.2	20.0	60.0
INMA, Spain-Gipuzkoa^*a*^
Children without autistic traits	340	50.3	10.0	36.5	53.5	2.4	32.8 ± 3.3	22.9 ± 3.4	163.7 ± 6.1	22.9	55.3
Children within the borderline or clinical range	17	47.1	35.3	35.3	29.4	5.9	33.0 ± 3.7	23.2 ± 4.6	162.2 ± 5.4	29.4	58.8
INMA, Spain-Sabadell^*a*^
Children without autistic traits	285	50.5	26.3	35.4	38.2	8.4	31.7 ± 4.1	23.6 ± 4.5	162.4 ± 5.8	27.0	60.7
Children within the borderline or clinical range	10	30.0	40.0	40.0	20.0	0.0	31.2 ± 5.0	22.7 ± 4.6	163.7 ± 6.7	30.0	50.0
INMA, Spain-Valencia
Children without autistic traits	484	49.8	26.7	43.2	30.2	7.9	31.9 ± 4.1	23.7 ± 4.4	162.2 ± 6.3	36.0	54.5
Children within the borderline or clinical range	37	24.3	43.2	51.4	5.4	10.8	30.6 ± 4.7	24.8 ± 4.4	162.0 ± 6.3	59.5	48.6
Children within the clinical range only	10	30.0	60.0	30.0	10.0	0.0	28.0 ± 2.4	24.9 ± 4.5	161.9 ± 6.6	60.0	50.0
^***a***^Because few children were classified as having autistic traits within the clinical range only in the Spanish cohorts of Gipuzkoa and Sabadell, we did not include that classification in the analysis.											

**Figure 1:**
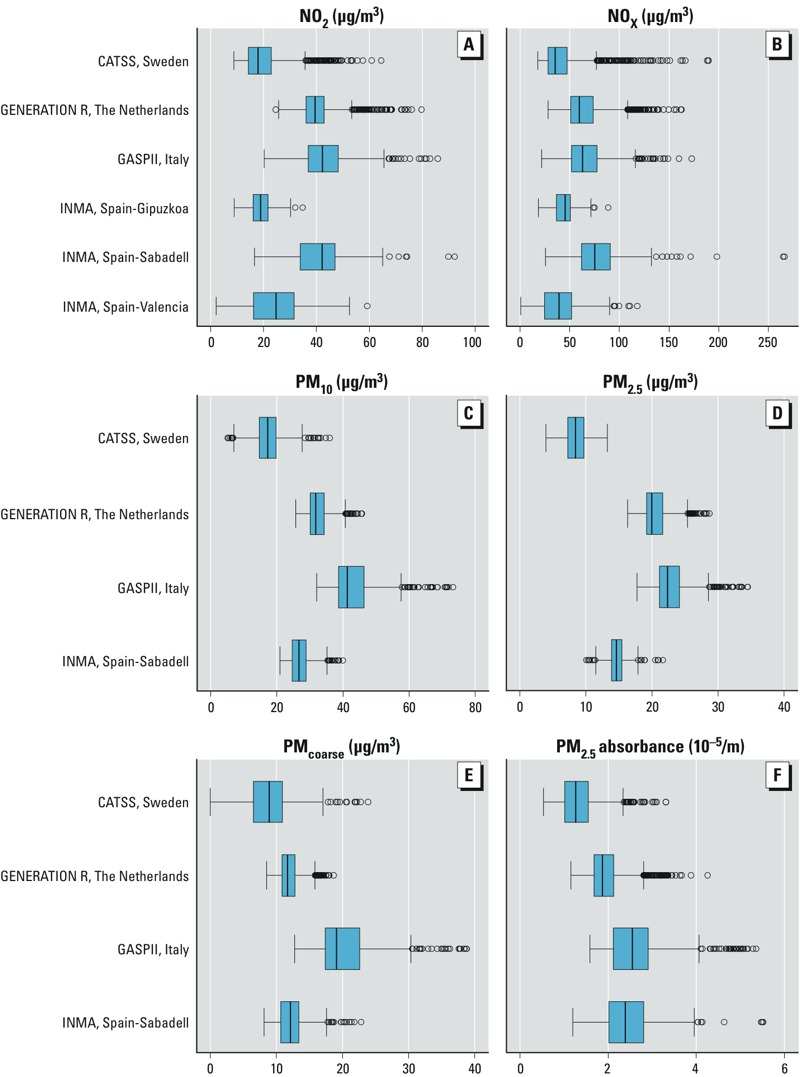
Distribution of air pollutant levels during pregnancy: (*A*) NO_2_; (*B*) NO_x_; (*C*) PM_10_; (*D*) PM_2.5_; (*E*) PM_coarse_; (*F*) PM_2.5_ absorbance. Air pollution levels were temporally adjusted to the exact pregnancy period. Boxes extend from the 25th to the 75th percentile, vertical bars represent the median, whiskers extend 1.5 times the length of the interquartile range above and below the 75th and 25th percentiles, respectively, and outliers are represented as points. PM_10_, PM_2.5_, PM_coarse_, and PM_2.5_absorbance were not available in the Spanish cohorts of Gipuzkoa and Valencia.

Median air pollution levels ranged from 17.9 μg/m^3^ (the Swedish cohort) to 42.2 μg/m^3^ (the Italian cohort) for NO_2_, and from 8.4 μg/m^3^ (the Swedish cohort) to 22.4 μg/m^3^ (the Italian cohort) for PM_2.5_ (Figure 1). Different correlation patterns between air pollution variables were found in the different cohorts (see Supplemental Material, Table S3). Overall, the correlation among air pollutants was strong (between 0.72 and 0.98), whereas the correlation between air pollutants and traffic variables was moderate or low (between 0.17 and 0.53).

**Figure 2:**
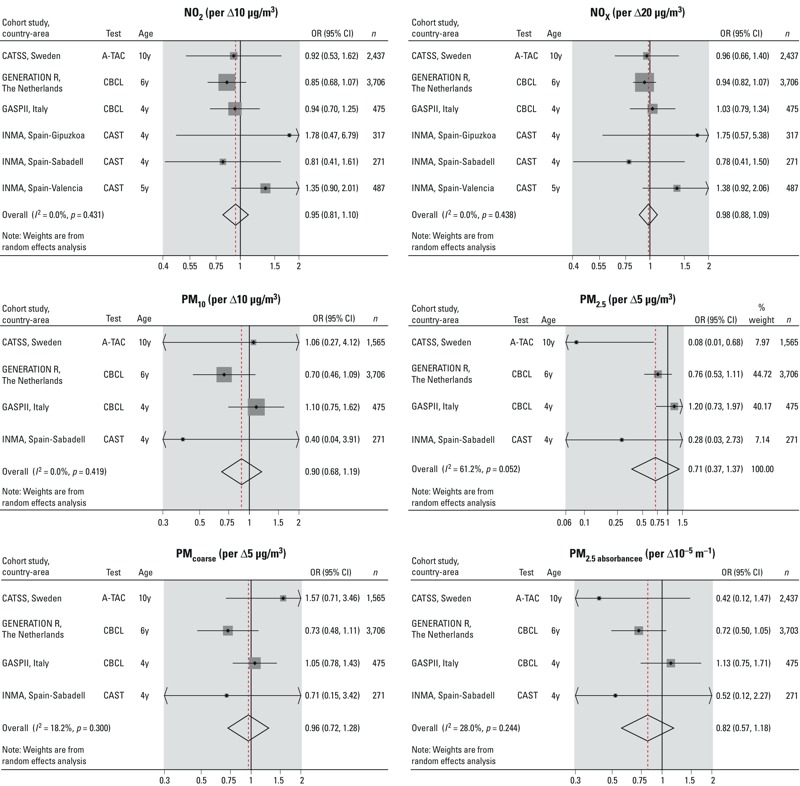
Fully adjusted associations between air pollution exposure during pregnancy and autistic traits within the borderline/clinical range. Coefficients and 95% confidence intervals by cohort and overall estimate were obtained by random-effects meta-analysis. Models were adjusted for maternal characteristics (education, country of birth, age at delivery, prepregnancy body mass index, height, prenatal smoking, parity), child’s sex, season at child’s birth, urbanicity at child’s birth address, child’s age (y, years) at the autistic traits assessment, and evaluator of the autistic traits. PM_10_, PM_2.5_, PM_coarse_, and PM_2.5 _absorbance were not available in the Spanish cohorts of Gipuzkoa and Valencia.

None of the air pollutants were associated with autistic traits within the borderline/clinical range in the minimally adjusted models [odds ratio (OR) = 1.02; 95% confidence interval (CI): 0.87, 1.19 per 10-μg/m^3^ increase average NO_2_ levels] (see Supplemental Material, Table S4). ORs changed only slightly in the fully adjusted models [changes of OR ranged from 0% (PM_10_) to 10% (PM_2.5_)] (Table 3). Fully adjusted associations of NO_2_ and children with autistic traits within the borderline/clinical range including all the potential confounding variables are shown in Supplemental Material, Table S5. As shown in Figure 2, in most cohorts the associations with the different pollutants were consistently close to one. However, for the Spanish cohorts of Valencia and Gipuzkoa, NO_2_ and NO_x_ exposure tended to have a slightly higher odds of autistic traits within the borderline/clinical range (Figure 2). Analysis with autistic traits within the clinical range (Table 3) and with autistic traits as quantitative scores (see Supplemental Material, Table S6) did not reveal any association with air pollution exposure.

**Table 3:** Fully adjusted combined associations*^a^* between air pollution during pregnancy*^b^* and autistic traits within the borderline/clinical range.

Pollutant	Autistic traits within the borderline/clinical range				Autistic traits within the clinical range			
	*n*^*c*^	OR (95% CI)	*p*-Heter	*I*^2^	*n*^*c*^	OR (95% CI)	*p*‑Heter	*I*^2^
NO_2_ (per Δ10 μg/m^3^)	6	0.95 (0.81, 1.10)	0.431	0.00%	4	0.87 (0.67, 1.14)	0.955	0.00%
NO_X_ (per Δ20 μg/m^3^)	6	0.98 (0.88, 1.09)	0.438	0.00%	4	0.93 (0.78, 1.11)	0.640	0.00%
PM_10_ (per Δ10 μg/m^3^)	4	0.90 (0.68, 1.19)	0.419	0.00%	3	0.92 (0.55, 1.54)	0.368	0.00%
PM_2.5_ (per Δ5 μg/m^3^)	4	0.71 (0.37, 1.37)	0.052	61.24%	3	1.01 (0.63, 1.63)	0.472	0.00%
PM_coarse_ (per Δ5 μg/m^3^)	4	0.96 (0.72, 1.28)	0.300	18.16%	3	0.87 (0.55, 1.38)	0.320	12.33%
PM_2.5_absorbance (per Δ10^–5^m^–1^)	4	0.82 (0.57, 1.18)	0.244	27.95%	3	0.70 (0.44, 1.12)	0.899	0.00%
Traffic intensity on the nearest road (per Δ5,000 mv/day)	3	1.00 (0.92, 1.09)	0.721	0.00%	3	0.98 (0.85, 1.14)	0.508	0.00%
Total traffic load on all major roads within 100-m buffer (per Δ4,000,000 mv/day × m)	5	1.02 (0.89, 1.16)	0.752	0.00%	3	0.90 (0.70, 1.16)	0.691	0.00%
Abbreviations: *I*^2^, percentage of the total variability due to between-areas heterogeneity; mv, motor vehicles; *p*-Heter, *p*-value of heterogeneity using the Cochran’s *Q* test. ^***a***^Odds ratios and 95% confidence intervals were estimated by random-effects meta-analysis by area. Models were adjusted for maternal characteristics (education, country of birth, age at delivery, prepregnancy body mass index, height, prenatal smoking, and parity), child’s sex, season at child’s birth, urbanicity at child’s birth address, and child’s age at autistic traits assessment, and evaluator of the autistic traits. Models of traffic variables were additionally adjusted for non–back-extrapolated background levels of NO_2_. ^***b***^Air pollution levels were temporally adjusted to the exact pregnancy period except for traffic variables. ^***c***^Number of cohorts included in the meta-analysis.								

We observed a similar lack of association in all sensitivity analyses: *a*) when cohorts were excluded one by one (see Supplemental Material, Table S7), *b*) when meta-analyses were restricted to cohorts with information on both NO_2_ and PM (see Supplemental Material, Table S8), *c*) when we used the 90th percentile of each autistic traits scale (see Supplemental Material, Table S9), *d*) when we stratified the meta-analyses by the type of evaluator (psychologist, parents) (see Supplemental Material, Table S10), *e*) when we assessed the non–back-extrapolated air pollution variables (see Supplemental Material, Table S11), *f*) when meta-analyses were restricted to children with postnatal stable residence (see Supplemental Material, Table S12), *g*) when meta-analyses were restricted to children of highly educated mothers (see Supplemental Material, Table S12) and children of mothers who did not smoke during pregnancy (see Supplemental Material, Table S12), and *h*) when meta-analyses were stratified by child’s sex (see Supplemental Material, Table S13).

## Discussion

In the present study we assessed the relationship between prenatal air pollution exposure including NO_2_ and PM and autistic traits in > 8,000 children of four European population-based birth/child cohorts. We found no evidence for an association between prenatal air pollution exposure and autistic traits in children 4–10 years of age. These results were consistent for all air pollutants assessed, across countries, using different cut-offs of autistic traits, examining autistic traits as continuous variables, and after adjusting for several socioeconomic status variables and urbanicity.

The strengths of our study were the large sample size in combination with the prospective and longitudinal study design, the use of a standardized and validated air pollution assessment in all countries, the assessment of exposure to a large number of air pollutants including NO_2_ and PM at the individual level, the assessment of autistic traits in childhood using standardized and validated neuropsychological tests, and the statistical analysis following a consensus protocol. Additionally, we adjusted for many socioeconomic and lifestyle variables known to be associated with air pollution exposure and/or autistic traits in children.

The main limitation of our study was that four different tests were used in the different cohorts to assess autistic traits. All four tests are valid tools for assessment of children’s behavior in epidemiological studies (Constantino and Gruber 2005; Larson et al. 2010; Sikora et al. 2008; Williams et al. 2005), though only two were developed to specifically address autistic traits (SRS and CAST). The other two (CBCL½–5 and A-TAC) have been most commonly used as screening tests for a broader range of behavioral profile and disorders including autistic traits. Although all tests include items corresponding to the three core features of ASD (social interaction deficits, communication deficits, and repetitive behaviors), each test includes a different number of items and gives a slightly different weight to each feature. These differences might imply that each instrument classifies children at risk for ASD in a slightly different way. However, children defined as having autistic traits within the borderline/clinical range or within the clinical range showed consistent associations with the assessed child and maternal characteristics across all cohorts (Table 2). Moreover, we found consistent null association between prenatal air pollution exposure and autistic traits among cohorts regardless of the type of instrument, the age of assessment, the type of evaluator of the test, and the treatment of the scales using different cut-offs or using it as a quantitative trait. Another limitation of our study is related to the exposure assessment. Air pollution levels were back-extrapolated to the pregnancy period, and this could lead to a nondifferential misclassification of the exposure. Air pollution campaigns were performed when children were between 3 and 10 years of age, depending on the cohort. We used long-term routine monitoring data for the back-extrapolation to the exact pregnancy period for which we assumed that the spatial distribution of the sources and predictors of air pollution levels were stable with time. Previous research supports this by showing a stability of measured and modeled spatial contrast in air pollutants over a period of 10 and 12 years (Cesaroni et al. 2012; Eeftens et al. 2011). However, because monitoring data were not available for all pollutants in all cohorts, particularly for PM, we used background monitoring network sites of other pollutants in the cases of missing information. Nevertheless, we found that back-extrapolated and non–back-extrapolated results were essentially similar. Because correlation between whole pregnancy and trimester-specific air pollution concentrations was high in a previous ESCAPE study (Pedersen et al. 2013), we did not attempt to calculate trimester-specific associations as these would not be expected to be different. Finally, paternal exposure to air pollution during the preconception phase may also play a role in the development of ASD, but this information was not available in our study.

Previous studies carried out in the United States found a consistently positive association between exposure to several air pollutants during pregnancy or during the first year of life and diagnosis of ASD (Becerra et al. 2013; Raz et al. 2015; Roberts et al. 2013; Volk et al. 2011, 2013; Windham et al. 2006). Moreover, a study carried out in Taiwan found that postnatal exposure to NO_2_, ozone, carbon monoxide, and sulfur dioxide was associated with ASD in children from 3 to 9 years of age (Jung et al. 2013). Results from previous studies seem contradictory to our findings. However, previous case–control studies selected children with a diagnosis of ASD, whereas in our study we studied children with autistic traits from population-based birth/child cohorts. A possible explanation for inconsistent findings could be that our study population does not represent the phenotypic extreme present in the case–control studies, because only a small number of children surpassed the threshold for ASD. We hypothesize that prenatal exposure to air pollution could be related to ASD but not associated with broad autistic traits in children. In our study we defined children with autistic traits within the clinical range using the clinical cut-off, which is shown to be specific for detecting children at risk for ASD, but results in small sample sizes. We found no indication of an association between prenatal air pollution exposure and autistic traits within the clinical range.

Residual confounding could be another possible explanation of the discrepant findings between previous studies and our study. As in our study, most of the previous studies accounted for several potential confounding variables including maternal education, maternal age, prenatal maternal smoking, and urbanicity. Becerra et al. (2013) found an association between ASD and air pollution exposure only after adjusting for maternal educational level, whereas associations were similar between unadjusted and adjusted models in the other studies (Volk et al. 2011, 2013). In our study, results were also materially unchanged after adjusting for confounders. Moreover, we observed similar results when we restricted our analyses to children of mothers with high educational levels or mothers who did not smoke during pregnancy. It is worth noting that children with diagnosis of ASD in some of the previous studies were more likely to come from high maternal socioeconomic backgrounds (Becerra et al. 2013; Kalkbrenner et al. 2010; Windham et al. 2006), whereas children with autistic traits in all our cohort studies were more likely to have mothers with a low maternal socioeconomic status. This finding may reflect a systematic difference between the study of broad autistic traits and children with ASD diagnosis, or that the previous studies share a common bias, possibly a diagnostic bias related to socioeconomic status–related differences in access to care, that was not operating in the European cohorts (Rai et al. 2012).

An alternative explanation of the contradictory findings could be differences in air pollution levels and sources. Air pollution levels at the extremes of the distributions in several of the previous studies are higher than those observed in our study, though exposure ranges do overlap [mean NO_2_ levels about 32–58 μg/m^3^ in California or Los Angeles county (Becerra et al. 2013; Volk et al. 2013) vs. 19–43 μg/m^3^ in our European cohorts; mean PM_10_ levels around 26–36 μg/m^3^ in California or Los Angeles county (Becerra et al. 2013; Volk et al. 2013) vs. 17–44 μg/m^3^ in our European cohorts; mean PM_2.5_ levels around 14–20 μg/m^3^ in California or Los Angeles (Becerra et al. 2013; Volk et al. 2013) county vs. 8–23 μg/m^3^ in our European cohorts]. In our study, NO_2_ and PM are markers of traffic air pollution, but also drive from sources such as space heating; small-scale traffic and population/household density variables were the most frequently used predictors in the land-use regression models (Beelen et al. 2013; Eeftens et al. 2012a). In some previous studies associations with ASD were also observed with traffic indicators such as living near a freeway or modeled traffic-related air pollution exposure (Volk et al. 2011, 2013). However, null association was observed in another study (Gong et al. 2014) when air pollutant levels were estimated using dispersion models. Although NO_2_ or PM levels are similar, different exposure mixtures among study areas may result in different health effects. Based on experimental models, it has been hypothesized that PM-soluble components are one of the major suspected culprits of the neurological effects of air pollution, primarily metals, because they may translocate from the respiratory tract into the systemic circulation and reach the fetus and promote oxidative stress and inflammation (Block et al. 2012). Further research including trace metal content of the PM, such as lead or manganese, is warranted to better understand the discrepant findings.

In conclusion, this study showed a null association between prenatal exposure to several air pollutants, including NO_2_ and PM, and autistic traits in children 4–10 years of age in four European population-based birth/child cohorts. Additional research including European studies of children with a diagnosis of ASD, a comparison of ASD manifestation and detection between the United States and Europe, and the study of the air pollutants mechanisms underlying the association with ASD is needed to further understand the different findings between previous studies and the present study.

## Supplemental Material

(923 KB) PDF
