# Supplementary material for: Air Pollution Exposure during Pregnancy and Childhood Autistic Traits in Four European Population-Based Cohort Studies: The ESCAPE Project
Source: Environ Health Perspect. 2015 Jun 12;124(1):133–40. doi: 10.1289/ehp.1408483 (PMC4710593; doi:10.1289/ehp.1408483)
Supplement: (923 KB) PDF [file ehp.1408483.s001.acco.pdf]

Environ Health Perspect
DOI: 10.1289/ehp.1408483

Note to Readers: *EHP* strives to ensure that all journal content is accessible to all readers. However, some figures and Supplemental Material published in *EHP* articles may not conform to 508 standards due to the complexity of the information being presented. If you need assistance accessing journal content, please contact ehp508@niehs.nih.gov. Our staff will work with you to assess and meet your accessibility needs within 3 working days.

Supplemental Material

Air Pollution Exposure during Pregnancy and Childhood Autistic Traits in Four European Population-Based Cohort Studies: The ESCAPE Project

Mònica Guxens, Akhgar Ghassabian, Tong Gong, Raquel Garcia-Esteban, Daniela Porta, Lise Giorgis-Allemand, Catarina Almqvist, Aritz Aranbarri, Rob Beelen, Chiara Badaloni, Giulia Cesaroni, Audrey de Nazelle, Marisa Estarlich, Francesco Forastiere, Joan Forns, Ulrike Gehring, Jesús Ibarluzea, Vincent W.V. Jaddoe, Michal Korek, Paul Lichtenstein, Mark J. Nieuwenhuijsen, Marisa Rebagliato, Rémy Slama, Henning Tiemeier, Frank C. Verhulst, Heather E. Volk, Göran Pershagen, Bert Brunekreef, and Jordi Sunyer

Table of Contents

Methods S1. Description of the air pollution assessment

Methods S2. Description of the autistic traits assessment

- Autism-Tics, Attention deficit and hyperactivity disorders, and other Comorbidities (A-TAC) inventory (Swedish cohort)
- Pervasive Developmental Problems (PDP) subscale of the Child Behavior Checklist for Toddlers (CBCL1½-5) (Dutch and Italian cohorts)
- Adapted 18-item version of the Social Responsiveness Scale (SRS) (Dutch cohort)
- Childhood Autism Spectrum Test (CAST) (Spanish cohort)

Table S1. Distribution of the autistic traits scales

Table S2. Power sample calculation

Table S3. Spearman correlations between air pollution levels during pregnancy and traffic indicator variables

Table S4. Minimally adjusted combined associations between air pollution exposure during pregnancy and autistic traits within the borderline/clinical range

Table S5. Fully adjusted associations between nitrogen dioxide exposure during pregnancy, potential confounding variables, and autistic traits within the borderline/clinical range across cohorts

Table S6. Fully adjusted associations between air pollution exposure during pregnancy and autistic traits as a quantitative trait across cohorts

Table S7. Fully adjusted combined associations between air pollution exposure during pregnancy and autistic traits within the borderline/clinical range, assessing the influence of a single cohort in the meta-analysis estimates

Table S8. Fully adjusted combined associations between nitrogen oxides exposure during pregnancy and autistic traits within the borderline/clinical range among cohorts with particulate matter variables available

Table S9. Fully adjusted combined associations between air pollution during pregnancy and autistic traits within the percentile 90th of each scale

Table S10. Fully adjusted combined associations between air pollution exposure during pregnancy and autistic traits within the borderline/clinical range stratified by type of evaluator of the test

Table S11. Fully adjusted combined associations between non-back-extrapolated air pollution exposure at child's birth address and autistic traits within the borderline/clinical range

Table S12. Sensitivity analyses of fully adjusted combined association between air pollution exposure during pregnancy and autistic traits within the borderline/clinical range

Table S13. Fully adjusted combined associations between air pollution exposure during pregnancy and autistic traits within the borderline/clinical range by child's sex

References

Methods S1. Description of the air pollution assessment

Air pollution concentrations at the participants' birth home addresses were estimated by Land-use regression models following a standardized procedure described elsewhere (Beelen et al. 2013; Eeftens et al. 2012a). Air pollution monitoring campaigns in the study areas were performed between October 2008 and January 2011. In all areas, three two-week measurements within one year of nitrogen dioxide (NO_2) and nitrogen oxides (NO_x) were performed at 80 sites (the Netherlands) or 40 sites (other areas) in the warm, cold, and intermediate seasons (Cyrys et al. 2013). In addition, in all cohorts except in the sub-cohorts of Valencia and Gipuzkoa from the Spanish cohort, simultaneous measurements of $PM_{2.5}$ absorbance (determined as the reflectance of $PM_{2.5}$ filters) and PM with aerodynamic diameters of less than 10μm (PM_{10}), less than 2.5μm ($PM_{2.5}$), and between 2.5 and 10μm (PM_{coarse}) were performed at half of the sites (Eeftens et al. 2012b). Results from the three measurements were then averaged, adjusting for temporal trends using data from a centrally located background monitoring site in each area. Predictor variables on nearby traffic intensity, population/household density, and land use were derived from Geographic Information Systems, and were evaluated to explain spatial variation of annual average concentrations using land-use regression. Land-use regression models were developed for each pollutant metric using all measurement sites, and in addition for background NO_2, using only rural and urban background sites. Land-use regression models were then used to estimate ambient air pollution concentration at the participants' birth home addresses, for which the same Geographic Information Systems predictor variables were collected. Moreover, we used a back-extrapolation procedure to estimate the concentrations back in time during each pregnancy of each woman I (Pedersen et al. 2013). The estimated yearly concentrations (Cyearly,i) at each home address i were combined with time-specific measurements from one centrally located background monitoring station by averaging the daily concentrations during

1) the year corresponding to the LUR yearly concentration (C_{yearly}) and 2) each pregnancy p_i considered (Cp_i). The ratio Cp_i/C_{yearly} constituted the temporal component of the model. For each pollutant, the concentration (Cp_{i}, i) estimated at the home address i during pregnancy for woman i was estimated as the product of the temporal (Cp_i/C_{yearly}) and spatial (C_{yearly}, i) components. If the monitoring station was in function for less than 75% of the pregnancy, we considered Cp_i as missing. In some cases, when air quality monitoring data from background station was unavailable for a given pollutant, we used measurements for another pollutant during the same time period as a replacement; the choice of that pollutant used to back-extrapolate another pollutant was based on an extensive study of temporal correlations between pollutants simultaneously available in each area (i.e. NO_X was used when $PM_{2.5}$ absorbance was missing (Swedish and Italian cohorts, and Sabadell sub-cohort of the Spanish cohort), PM_{10} was used when $PM_{2.5}$ was missing (Dutch and Italian cohorts), $PM_{2.5}$ was used when PM_{10} was missing (Swedish cohort), NO_2 when PM_{10} was missing (Sabadell sub-cohort of the Spanish cohort)). We accounted for change of home address during pregnancy in estimation of exposure when the date of moving and new address was available (Dutch cohort). In addition to predicted concentrations, some cohorts were able to collect traffic intensity on the nearest road (Swedish, Dutch, and Italian cohorts) and total traffic load (intensity*length) on all major roads within a 100m buffer (Swedish, Dutch and Italian cohorts, and the Spanish Valencia and Sabadell cohorts).

Methods S2. Description of the autistic traits assessment

Autism-Tics, Attention deficit and hyperactivity disorders, and other Comorbidities (A-TAC) inventory (Swedish cohort)

The A-TAC inventory is a parental telephone interview designed for large-scale epidemiological research that covers a broad range of neurodevelopmental disorders (Anckarsäter et al. 2011; Larson et al. 2010). The short version consists of 96 questions

divided into several problem areas worded to reflect Diagnostic and Statistical Manual of Mental Disorders, 4th edition (DSM-IV) criteria (APA 2000) and clinical features. For the present study we used the autism spectrum disorder module that consists of 17 questions. Questions are answered from a lifetime perspective and scores for single items are coded as 0 for "no," 0.5 for "yes, to some extent," and 1 for "yes." Seventeen item scores were added to form a sum score measuring the resemblance to the clinical diagnose of autism spectrum disorders. Higher scores indicate more autistic traits. Two validation studies showed that A-TAC is a sensitive tool to screen for autism spectrum disorders (Hansson et al. 2005; Larson et al. 2010). Cut-offs to yield proxies for autistic traits within the borderline or clinical range (at 4.5 points that correspond to the highest possible cut-off that yielded a sensitivity ≥0.95) and within the clinical range (at 8.5 points that correspond to the lowest cut-off that yielded a specificity ≥0.95) were established (Anckarsäter et al. 2011).

Pervasive Developmental Problems (PDP) subscale of the Child Behavior Checklist for Toddlers (CBCL1½-5) (Dutch and Italian cohorts)

The CBCL1½-5 is a highly validated instrument to measure parental-reported behavioral and emotional problems of children at young age (Achenbach and Rescorla 2000). The Dutch and the Italian versions are reliable and well validated (Muratori et al. 2011; Tick et al. 2007), and the subscales for syndromes derived from the CBCL1½-5 had good fit in 23 international studies across diverse societies (Ivanova et al. 2010), and are consistent with diagnostic categories of the Diagnostic and Statistical Manual of Mental Disorders, 4th edition (DSM-IV) (APA 2000). The pervasive developmental problems (PDP) subscale, a DSM-oriented scale that aims to identify children at risk for autism spectrum disorders, consists of 13 items. Each item of the questionnaire describes a specific behavior and the parent is asked to rate its frequency on a three point Likert scale (0, not true; 1, somewhat or sometimes true; 2, very true or often true). Higher scores indicate more autistic traits. The PDP subscale has a good

predictive validity to identify children at risk of autism spectrum disorders (Sikora et al. 2008), with areas under the receiving operating characteristic (ROC) curve of 0.95 (Muratori et al 2011). We used the 93rd and 98th percentiles of a Dutch norm group as cutoff scores to classify children with autistic traits within the borderline or clinical range and the clinical range, respectively (Tick et al. 2007).

Adapted 18-item version of the Social Responsiveness Scale (SRS) (Dutch cohort)

The SRS is a parental-reported questionnaire designed to assess autistic traits for children between 4-18 years of age as a quantitative trait (Constantino and Gruber 2005; Constantino and Todd 2000). In order to minimize subject burden, the lengthy original questionnaire was reduced to an adapted 18-item version of the SRS (Román et al. 2013). Items selected encompassed all DSM-IV autism domains (social cognition, social communication, and autistic mannerism) and were chosen based on the personal communication with the developer of the SRS (Román et al. 2013). In our study, the Crohnbach's alpha indicated high inter-item reliability for the adapted 18-item version of the SRS (alpha=0.79). The adapted 18-item version of the SRS correlated well with the pervasive developmental problems scale (r=0.59, p<.001). In a sample of 3,857 children aged 4-18 years (as part of the Social Spectrum Study, a multicenter study social development in the children referred to a mental health care institution in the South-West of the Netherlands from 2010-2012) the correlation between total scores derived from the selected 18 items and the SRS scores derived from the complete test was r=0.95 (p<0.001) (data not published, provided by AG). Moreover, in a sample of 2,719 children from the Interactive Autism Network's the correlation between total scores derived from the selected 18 items and from the complete SRS was r=0.99 (p<0.001) (data not published, provided by AG). Each item is rated from 0 (never true) to 3 (almost always true), covering social, language, and repetitive behaviors. Higher scores indicate more

autistic traits. Since borderline and clinical cut-offs are not defined for the adapted 18-item version of the SRS, we only analyzed that scale as a quantitative trait.

Childhood Autism Spectrum Test (CAST) (Spanish cohort)

The CAST is a questionnaire administered to the parents by a psychologist based on behavioral descriptions of the ICD-10 (WHO 1993) and Diagnostic and Statistical Manual of Mental Disorders, 4th edition (DSM-IV) criteria (APA 2000) designed to identify subtle manifestations of autism spectrum conditions (social impairments, communication impairments and repetitive or stereotyped behaviors) (Baron-Cohen et al. 2009). The questionnaire includes 31 key items coded as “No” or ”Yes” that contribute to a child’s total score, along with 6 control questions on general development. Higher scores indicate more autistic traits. The CAST is shown to have high sensitivity in studies with primary school age children in mainstream schools (Williams et al. 2005). Cut-offs to yield proxies for autistic traits within the borderline or clinical range (at 12 points that correspond to a sensitivity ≥0.99) and within the clinical range (at 15 points that correspond to a specificity ≥0.97) were established (Williams et al. 2005).

Table S1. Distribution of the autistic traits scales

Cohort study	Test	Range	P_{10}	P_{25}	P_{50}	P_{75}	P_{90}	Mean ± SD	Cut-off borderline or clinical range	Cut-off clinical range only
CATSS, Sweden	A-TAC	0 to 16	0	0	0	1	2	0.77 ± 1.56	4.5	8.5
GENERATION R, the Netherlands	PDP subscale (CBCL½-5)	0 to 22	0	0	2	3	5	2.19 ± 2.46	6	8
	SRS	0 to 45	0	1	3	5	8	3.91 ± 4.25	na[a]	na[a]
GASPII, Italy	PDP subscale (CBCL½-5)	0 to 22	0	1	2	4	6	2.61 ± 2.37	6	8
INMA, Spain-Gipuzkoa	CAST	0 to 18	2	4	5	8	10	5.75 ± 3.22	12	15
INMA, Spain-Sabadell	CAST	0 to 15	2	3	4	7	9	5.01 ± 2.89	12	15
INMA, Spain-Valencia	CAST	0 to 19	2	4	6	8	11	6.21 ± 3.21	12	15

A-TAC, autism-tics, attention deficit and hyperactivity disorders, and other comorbidities inventory; CBCL, child behavior checklist; CAST, childhood autism spectrum test; na, not applicable; PDP, pervasive developmental problems; SD, standard deviation; SRS, Social Responsiveness Scale

[a]na=not applicable since the cut-off points for autistic traits within the borderline and clinical ranges have not been defined for the 18-item version of the SRS, score only evaluated as a continuous quantitative outcome

Table S2. Power sample calculation

Accepting a type I error of 5% in a two-sided test, we had an 80% power to detect the following ORs:

**Air pollutant**	**Borderline or clinical range**	**Clinical range**
NO_2 (per Δ10 μg/m^3)	1.14	1.22
NO_X (per Δ20 μg/m^3)	1.14	1.29
PM_{10} (per Δ10 μg/m^3)	1.14	1.27
$PM_{2.5}$ (per Δ5 μg/m^3)	1.14	1.26
PM_{coarse} (per Δ5 μg/m^3)	1.15	1.27
$PM_{2.5}$absorbance (per Δ10^{-5}m^{-1})	1.14	1.27
Traffic intensity on the nearest road (per Δ5,000 mv/day)	1.13	1.27
Total traffic load on all major roads within 100m buffer (per Δ4,000,000 mv/day*m)	1.14	1.27

Variations in sample sizes for the different exposures and outcomes were taken into account

Table S3. Spearman correlations between air pollution levels during pregnancy[a] and traffic indicator variables

Cohort study	NO_2 *vs.* NOx	NO_2 *vs.* $PM_{2.5}$ absorbance	NO_x *vs.* $PM_{2.5}$ absorbance	$PM_{2.5}$ *vs.* $PM_{2.5}$ absorbance	NO_2 *vs.* Traffic intensity	NO_2 *vs.* Traffic load	$PM_{2.5}$ absorbance *vs.* Traffic intensity	$PM_{2.5}$ absorbance *vs.* Traffic load
CATSS, Sweden	0.896*	0.844*	0.783*	0.546*	0.464*	0.401*	0.340*	0.333*
GENERATION R, the Netherlands	0.859*	0.803*	0.783*	0. 739*	0.257*	0.327*	0.192*	0.290*
GASPII, Italy	0.722*	0.600*	0.772*	0.735*	0.172*	0.457*	0.453*	0.527*
INMA, Spain-Gipuzkoa	0.960*	na	na	na	na	na	na	na
INMA, Spain-Sabadell	0.894*	0.769*	0.953*	0.760*	na	0.439*	na	0.460*
INMA, Spain-Valencia	0.981*	na	na	na	na	0.310*	na	na

na, data not available; PM2.5, particle matter less than 2.5µm; $PM_{2.5}$absorbance, reflectance of $PM_{2.5}$ filters; Traffic intensity, traffic intensity on the nearest road; Traffic load, total traffic load (intensity*length) on all major roads within 100m buffer.

[a]Air pollution levels were temporally adjusted to the exact pregnancy period except for the traffic indicator variables

*p-value <0.05

Table S4. Minimally adjusted combined associations[a] between air pollution exposure during pregnancy[b] and autistic traits within the borderline/clinical range

Air pollutant	N[c]	OR	(95% CI)	p-heter	I^2
NO_2 (per Δ10 μg/m^3)	6	1.02	(0.87, 1.19)	0.304	16.97%
NO_X (per Δ20 μg/m^3)	6	1.04	(0.90, 1.19)	0.225	28.02%
PM_{10} (per Δ10 μg/m^3)	4	0.90	(0.67, 1.20)	0.347	9.14%
$PM_{2.5}$ (per Δ5 μg/m^3)	4	0.79	(0.48, 1.31)	0.111	50.09%
PM_{coarse} (per Δ5 μg/m^3)	4	0.95	(0.73, 1.23)	0.312	15.85%
$PM_{2.5}$absorbance (per Δ10^{-5}m^{-1})	4	0.86	(0.62, 1.21)	0.198	35.65%
Traffic intensity on the nearest road (per Δ5,000 mv/day)	3	0.99	(0.91, 1.07)	0.413	0.00%
Total traffic load on all major roads within 100m buffer (per Δ4,000,000 mv/day*m)	5	0.98	(0.86, 1.12)	0.875	0.00%

95% CI, 95% Confidence Interval; OR, Odds Ratio; I^2 =Percentage of the total variability due to between-cohorts heterogeneity; NO_2, nitrogen dioxide; NO_x, nitrogen oxides; p-heter, P value of heterogeneity using the Cochran's Q test; PM_{10}, particle matter less than 10μm; $PM_{2.5}$, particle matter less than 2.5μm; PM_{coarse}, particle matter between 2.5 and 10μm; $PM_{2.5}$absorbance, reflectance of $PM_{2.5}$ filters

[a] Odds Ratio and 95% confidence interval estimated by random-effects meta-analysis by area. Models were adjusted for child's sex and child's age at autistic traits assessment. Models of traffic indicator variables were additionally adjusted for non-back-extrapolated background levels of NO_2

[b] Air pollution levels were temporally adjusted to the exact pregnancy period, except for the traffic indicator variables

[c] Number of cohorts included in the meta-analysis

Table S5. Fully adjusted associations between nitrogen dioxide exposure during pregnancy[a], potential confounding variables, and autistic traits within the borderline/clinical range across cohorts

	CATSS, Sweden		GENERATION R, the Netherlands		GASPII, Italy		INMA, Spain-Gipuzkoa		INMA, Spain-Sabadell		INMA, Spain-Valencia	
**Variables**	**OR**	**(95% CI)**	**OR**	**(95% CI)**	**OR**	**(95% CI)**	**OR**	**(95% CI)**	**OR**	**(95% CI)**	**OR**	**(95% CI)**
NO_2 (per Δ10 μg/m^3)	0.92	(0.53, 1.62)	0.85	(0.68, 1.07)	0.94	(0.70, 1.25)	1.78	(0.47, 6.79)	0.81	(0.41, 1.61)	1.35	(0.90, 2.01)
Maternal educational level: Low	1.00		1.00		1.00		1.00		1.00		1.00	
Medium	0.39	(0.13, 1.17)	0.59	(0.38, 0.92)	0.42	(0.19, 0.96)	0.32	(0.09, 1.14)	0.64	(0.13, 3.08)	0.87	(0.40, 1.93)
High	0.12	(0.03, 0.46)	0.53	(0.33, 0.85)	0.31	(0.13, 0.77)	0.18	(0.05, 0.68)	0.32	(0.05, 2.20)	0.17	(0.04, 0.82)
Maternal country of birth (foreign vs. non-foreign)	0.32	(0.07, 1.36)	1.39	(1.05, 1.84)	na		2.11	(0.18, 27.56)	na		1.70	(0.46, 6.30)
Maternal age at delivery (years)	0.95	(0.87, 1.03)	0.98	(0.95, 1.01)	1.02	(0.95, 1.09)	1.02	(0.87, 1.21)	0.96	(0.81, 1.15)	0.92	(0.83, 1.01)
Maternal pre-pregnancy body mass index (kg/m^2)	1.08	(0.98, 1.19)	0.99	(0.96, 1.02)	1.08	(1.00, 1.16)	1.00	(0.87, 1.15)	0.92	(0.76, 1.10)	1.04	(0.97, 1.12)
Maternal height (cm)	1.01	(0.95, 1.07)	1.00	(0.98, 1.01)	1.00	(0.95, 1.05)	0.97	(0.89, 1.06)	1.04	(0.92, 1.19)	1.02	(0.96, 1.09)
Maternal smoking during pregnancy (yes vs. no)	2.00	(0.75, 5.37)	1.38	(1.00, 1.91)	0.54	(0.19, 1.51)	1.05	(0.31, 3.56)	0.98	(0.22, 4.47)	2.52	(1.17, 5.43)
Parity: Nulliparous	1.00		1.00		1.00		1.00		1.00		1.00	
1 child	0.40	(0.19, 0.85)	0.69	(0.52, 0.92)	0.36	(0.19, 0.70)	0.73	(0.23, 2.33)	1.95	(0.41, 9.17)	1.20	(0.52, 2.78)
≥2 children	0.49	(0.20, 1.19)	0.62	(0.39, 0.97)	0.33	(0.07, 1.62)	0.80	(0.07, 8.75)	2.14	(0.13, 34.58)	2.19	(0.57, 8.47)
Urbanicity at child's birth address (rural vs. urban)	0.40	(0.16, 0.99)	na		na		na		na		0.73	(0.13, 4.05)
Child's sex (female vs. male)	0.19	(0.08, 0.42)	0.52	(0.41, 0.67)	0.64	(0.36, 1.12)	0.73	(0.26, 2.04)	0.35	(0.08, 1.49)	0.33	(0.14, 0.77)
Season at child's birth date:												
Winter	1.00		1.00		1.00		1.00		1.00		1.00	
Spring	1.33	(0.44, 4.02)	1.31	(0.91, 1.89)	0.86	(0.36, 2.04)	1.18	(0.30, 4.61)	0.33	(0.03, 4.15)	0.35	(0.10, 1.19)
Summer	1.20	(0.39, 3.68)	1.16	(0.82, 1.64)	0.61	(0.24, 1.55)	0.82	(0.20, 3.39)	1.27	(0.20, 8.20)	0.53	(0.20, 1.39)
Autumn	1.28	(0.42, 3.89)	1.04	(0.74, 1.47)	0.40	(0.16, 1.00)	1.20	(0.26, 5.57)	0.88	(0.13, 5.83)	0.35	(0.13, 0.90)
	0.89[c]	(0.77, 1.01)	0.86	(0.63, 1.16)	0.63	(0.15, 2.58)	0.71	(0.00, 145.9)	2.46	(0.07, 83.2)	1.71	(0.23, 12.66)
Age at autistic traits assessment[b]	1.05[d]	(0.99, 1.10)										
Evaluator of the autistic traits	na		na		na		na		na		0.49	(0.19, 1.24)

[a]Air pollution levels were temporally adjusted to the exact pregnancy period

[b]All age-relationships were linear except for the Swedish cohort where the best fit fractional polynomial of degree 2 had powers (3[c],3[d])

Table S6. Fully adjusted[a] associations between air pollution exposure during pregnancy[b] and autistic traits as a quantitative trait across cohorts

	NO_2 (per Δ10 μg/m^3)			NO_X (per Δ20 μg/m^3)		
**Cohort study**	**n**	**MR**	**(95% CI)**	**n**	**MR**	**(95% CI)**
CATSS, Sweden	2,437	0.96	(0.86, 1.08)	2,437	0.98	(0.90, 1.06)
GENERATION R, the Netherlands (CBCL test)	3,706	0.97	(0.91, 1.04)	3,706	1.00	(0.96, 1.04)
GENERATION R, the Netherlands (SRS test)	3,036	0.92	(0.86, 0.97)	3,036	0.96	(0.93, 1.00)
GASPII, Italy	494	0.94	(0.87, 1.02)	494	0.96	(0.89, 1.03)
INMA, Spain-Gizpukoa	355	1.07	(0.92, 1.24)	355	1.07	(0.94, 1.21)
INMA, Spain-Sabadell	295	0.95	(0.90, 1.02)	295	0.96	(0.91, 1.01)
INMA, Spain-Valencia	487	1.03	(0.99, 1.08)	487	1.04	(0.99, 1.09)

	PM_{10} (per Δ10 μg/m^3)			$PM_{2.5}$ (per Δ5 μg/m^3)		
**Cohort study**	**n**	**MR**	**(95% CI)**	**n**	**MR**	**(95% CI)**
CATSS, Sweden	1,565	0.97	(0.76, 1.25)	1,565	0.77	(0.55, 1.09)
GENERATION R, the Netherlands (CBCL test)	3,706	0.95	(0.85, 1.08)	3,706	0.96	(0.86, 1.06)
GENERATION R, the Netherlands (SRS test)	3,036	0.84	(0.75, 0.94)	3,036	0.87	(0.78, 0.96)
GASPII, Italy	494	0.95	(0.85, 1.06)	494	0.94	(0.81, 1.08)
INMA, Spain-Sabadell	295	0.85	(0.70, 1.03)	295	0.73	(0.59, 0.90)

	PM_{coarse} (per Δ5 μg/m^3)			$PM_{2.5}$absorbance (per Δ10^{-5}m^{-1})		
**Cohort study**	**n**	**MR**	**(95% CI)**	**n**	**MR**	**(95% CI)**
CATSS, Sweden	1,565	1.02	(0.89, 1.18)	2,437	0.76	(0.59, 0.98)
GENERATION R, the Netherlands (CBCL test)	3,706	0.97	(0.86, 1.09)	3,703	0.92	(0.83, 1.02)
GENERATION R, the Netherlands (SRS test)	3,036	0.86	(0.77, 0.96)	3,033	0.83	(0.75, 0.92)
GASPII, Italy	494	0.96	(0.88, 1.05)	494	0.95	(0.85, 1.07)
INMA, Spain-Sabadell	295	0.96	(0.84, 1.10)	295	0.89	(0.79, 1.00)

95% CI, 95% Confidence Interval; I^2 =Percentage of the total variability due to between-cohorts heterogeneity; MR, Mean Ratio; NO_2, nitrogen dioxide; NO_x, nitrogen oxides; p-heter, P value of heterogeneity using the Cochran's Q test; PM_{10}, particle matter less than 10μm; $PM_{2.5}$, particle matter less than 2.5μm; PM_{coarse}, particle matter between 2.5 and 10μm; $PM_{2.5}$absorbance, reflectance of $PM_{2.5}$ filters

[a]Mean Ratio and 95% confidence interval estimated by negative binomial regression. Models were adjusted for maternal education, maternal country of birth, maternal age at delivery, maternal pre-pregnancy body mass index, maternal height, maternal smoking during pregnancy, parity, child's sex, season at child's birth date, urbanicity at child's birth address, child's age at autistic traits assessment, and type of evaluator of the autistic traits test

[b]Air pollution levels were temporally adjusted to the exact pregnancy period

Table S7. Fully adjusted combined associations[a] between air pollution exposure during pregnancy[b] and autistic traits within the borderline/clinical range, assessing the influence of a single cohort in the meta-analysis estimates

	NO_2 (per Δ10 µg/m^3)		NO_X (per Δ20 µg/m^3)		PM_{10} (per Δ10 µg/m^3)		$PM_{2.5}$ (per Δ5 µg/m^3)		PM_{coarse} (per Δ5 µg/m^3)		$PM_{2.5}$absorbance (per Δ$10^{-5}m^{-1}$)	
**Cohort study**	**OR**	**(95% CI)**	**OR**	**(95% CI)**	**OR**	**(95% CI)**	**OR**	**(95% CI)**	**OR**	**(95% CI)**	**OR**	**(95% CI)**
Combined estimate	0.95	(0.81, 1.10)	0.98	(0.88, 1.09)	0.90	(0.68, 1.19)	0.71	(0.37, 1.37)	0.96	(0.72, 1.28)	0.82	(0.57, 1.18)
Cohorts omited[c]:												
CATSS, Sweden	0.96	(0.80, 1.16)	1.01	(0.87, 1.17)	0.88	(0.60, 1.27)	0.89	(0.58, 1.35)	0.92	(0.71, 1.18)	0.87	(0.60, 1.25)
GENERATION R, the Netherlands	1.03	(0.84, 1.26)	1.07	(0.89, 1.28)	1.07	(0.74, 1.54)	0.38	(0.07, 2.24)	1.09	(0.83, 1.44)	0.82	(0.43, 1.56)
GASPII, Italy	0.98	(0.78, 1.22)	1.00	(0.85, 1.17)	0.72	(0.48, 1.08)	0.35	(0.09, 1.43)	0.91	(0.54, 1.54)	0.68	(0.48, 0.97)
INMA, Spain-Gipuzkoa	0.94	(0.81, 1.09)	0.98	(0.88, 1.09)	NA		NA		NA		NA	
INMA, Spain-Sabadell	0.97	(0.81, 1.16)	1.00	(0.88, 1.13)	0.91	(0.66, 1.25)	0.76	(0.38, 1.52)	0.98	(0.69, 1.38)	0.83	(0.54, 1.27)
INMA, Spain-Valencia	0.89	(0.76, 1.05)	0.96	(0.86, 1.07)	NA		NA		NA		NA	

95% CI, 95% Confidence Interval; OR, Odds Ratio; NO_2, nitrogen dioxide; NO_x, nitrogen oxides; PM_{10}, particle matter less than 10µm; $PM_{2.5}$, particle matter less than 2.5µm; PM_{coarse}, particle matter between 2.5 and 10µm; $PM_{2.5}$absorbance, reflectance of $PM_{2.5}$ filters; NA, Not Available.

[a]Odds Ratios and 95% confidence interval estimated by random-effects meta-analysis by area. Models were adjusted for maternal education, maternal country of birth, maternal age at delivery, maternal pre-pregnancy body mass index, maternal height, maternal smoking during pregnancy, parity, child's sex, urbanicity at child’s birth address, child's age at autistic traits assessment, and type of evaluator of the autistic traits test

[b]Air pollution levels were temporally adjusted to the exact pregnancy period

[c]Meta-analysis estimates computed omitting one cohort in each the time

Table S8. Fully adjusted combined associations[a] between nitrogen oxides exposure during pregnancy[b] and autistic traits within the borderline/clinical range among cohorts with particulate matter variables available

	NO_2 (per Δ10 µg/m^3)		NO_X (per Δ20 µg/m^3)	
**Cohort study**	**OR**	**(95% CI)**	**OR**	**(95% CI)**
CATSS, Sweden	0.92	(0.53, 1.62)	0.96	(0.66, 1.40)
GENERATION R, the Netherlands	0.85	(0.68, 1.07)	0.94	(0.82, 1.07)
GASPII, Italy	0.94	(0.70, 1.25)	1.03	(0.79, 1.34)
INMA, Spain-Sabadell	0.81	(0.41, 1.61)	0.78	(0.41, 1.50)
Combined estimate	0.88	(0.75, 1.04)	0.95	(0.85, 1.06)
p-heter		0.950		0.858
I^2		0.00		0.00

95% CI, 95% Confidence Interval; OR, Odds Ratio; I^2 =Percentage of the total variability due to between-cohorts heterogeneity; NO_2, nitrogen dioxide; NO_x, nitrogen oxides; p-heter, P value of heterogeneity using the Cochran's Q test

[a]Odds Ratios and 95% confidence interval estimated by random-effects meta-analysis by area. Models were adjusted for maternal education, maternal country of birth, maternal age at delivery, maternal pre-pregnancy body mass index, maternal height, maternal smoking during pregnancy, parity, child's sex, urbanicity at child's birth address, child's age at autistic traits assessment, and type of evaluator of the autistic traits test

[b]Air pollution levels were temporally adjusted to the exact pregnancy period

Table S9. Fully adjusted combined associations[a] between air pollution during pregnancy[b] and autistic traits within the percentile 90th of each scale

Air pollutant	N[c]	OR	(95% CI)	p-heter	I^2
NO_2 (per D10 μg/m^3)	6	0.94	(0.81, 1.10)	0.367	7.78%
NO_X (per D20 μg/m^3)	6	0.97	(0.86, 1.11)	0.284	19.77%
PM_{10} (per D10 μg/m^3)	4	0.80	(0.60, 1.07)	0.699	0.00%
$PM_{2.5}$ (per D5 μg/m^3)	4	0.69	(0.50, 0.95)	0.378	2.91%
PM_{coarse} (per D5 μg/m^3)	4	0.93	(0.73, 1.19)	0.321	14.18%
$PM_{2.5}$absorbance (per D10^{-5}m^{-1})	4	0.69	(0.49, 0.97)	0.255	26.05%
Traffic intensity on the nearest road (per Δ5,000 mv/day)	3	1.03	(0.95, 1.11)	0.585	0.00%
Total traffic load on all major roads within 100m buffer (per Δ4,000,000 mv/day*m)	5	0.98	(0.86, 1.12)	0.755	0.00%

95% CI, 95% Confidence Interval; OR, Odds Ratio; I^2 =Percentage of the total variability due to between-cohorts heterogeneity; NO_2, nitrogen dioxide; NO_x, nitrogen oxides; p-heter, P value of heterogeneity using the Cochran's Q test; PM_{10}, particle matter less than 10μm; $PM_{2.5}$, particle matter less than 2.5μm; PM_{coarse}, particle matter between 2.5 and 10μm; $PM_{2.5}$absorbance, reflectance of $PM_{2.5}$ filters

[a] Odds Ratio and 95% confidence interval estimated by random-effects meta-analysis by area. Models were adjusted for maternal education, maternal country of birth, maternal age at delivery, maternal pre-pregnancy body mass index, maternal height, maternal smoking during pregnancy, parity, child's sex, season at child's birth date, urbanicity at child's birth address, child's age at autistic traits assessment, and type of evaluator of the autistic traits test. Models of traffic indicator variables were additionally adjusted for non-back-extrapolated background levels of NO_2

[b]Air pollution levels were temporally adjusted to the exact pregnancy period except for the traffic indicator variables

[c]Number of cohorts included in the meta-analysis

Table S10. Fully adjusted combined associations[a] between air pollution exposure during pregnancy[b] and autistic traits within the borderline/clinical range stratified by type of evaluator of the test

	Parents					Psychologist				
**Air pollutant**	**N[c]**	**OR**	**(95% CI)**	**p-heter**	**I^2**	**N[c]**	**OR**	**(95% CI)**	**p-heter**	**I^2**
NO_2 (per Δ10 μg/m^3)	3	0.89	(0.75, 1.05)	0.871	0.00%	3	1.21	(0.87, 1.69)	0.386	0.00%
NO_X (per Δ20 μg/m^3)	3	0.96	(0.85, 1.07)	0.816	0.00%	3	1.20	(0.80, 1.78)	0.282	20.92%
PM_{10} (per Δ10 μg/m^3)	3	0.91	(0.66, 1.25)	0.312	14.19%	1	0.40	(0.04, 3.91)	na	na
$PM_{2.5}$ (per Δ5 μg/m^3)	3	0.76	(0.38, 1.52)	0.033	70.73%	1	0.28	(0.03, 2.73)	na	na
PM_{coarse} (per Δ5 μg/m^3)	3	0.98	(0.69, 1.38)	0.172	43.16%	1	0.71	(0.15, 3.42)	na	na
$PM_{2.5}$absorbance (per Δ10^{-5}m^{-1})	3	0.83	(0.54, 1.27)	0.153	46.66%	1	0.52	(0.12, 2.27)	na	na
Traffic intensity on the nearest road (per Δ5,000 mv/day)	3	1.00	(0.92, 1.09)	0.721	0.00%	0	na	na	na	na
Total traffic load on all major roads within 100m buffer (per Δ4,000,000 mv/day*m)	3	0.99	(0.84, 1.15)	0.508	0.00%	2	1.10	(0.85, 1.42)	0.807	0.00%

95% CI, 95% Confidence Interval; OR, Odds Ratio; I^2 =Percentage of the total variability due to between-cohorts heterogeneity; NO_2, nitrogen dioxide; NO_x, nitrogen oxides; p-heter, P value of heterogeneity using the Cochran's Q test; PM_{10}, particle matter less than 10μm; $PM_{2.5}$, particle matter less than 2.5μm; PM_{coarse}, particle matter between 2.5 and 10μm; $PM_{2.5}$absorbance, reflectance of $PM_{2.5}$ filters; NA, not applicable

[a] Odds Ratio and 95% confidence interval estimated by random-effects meta-analysis by area. Models were adjusted for maternal education, maternal country of birth, maternal age at delivery, maternal pre-pregnancy body mass index, maternal height, maternal smoking during pregnancy, parity, child's sex, season at child's birth date, urbanicity at child's birth address, and child's age at autistic traits assessment. Models of traffic indicator variables were additionally adjusted for non-back-extrapolated background levels of NO_2

[b]Air pollution levels were temporally adjusted to the exact pregnancy period except for the traffic indicator variables

[c]Number of cohorts included in the meta-analysis

Table S11. Fully adjusted combined associations[a] between non-back-extrapolated air pollution exposure at child's birth address and autistic traits within the borderline/clinical range

Air pollutant	N[b]	OR	(95% CI)	p-heter	I^2
NO_2 (per Δ10 µg/m^3)	6	0.96	(0.82, 1.13)	0.509	0.00%
NO_X (per Δ20 µg/m^3)	6	0.99	(0.87, 1.12)	0.614	0.00%
PM_{10} (per Δ10 µg/m^3)	4	0.81	(0.56, 1.19)	0.498	0.00%
$PM_{2.5}$ (per Δ5 µg/m^3)	4	0.68	(0.36, 1.29)	0.247	27.43%
PM_{coarse} (per Δ5 µg/m^3)	4	0.89	(0.67, 1.17)	0.865	0.00%
$PM_{2.5}$absorbance (per Δ10^{-5}m^{-1})	4	0.79	(0.48, 1.31)	0.195	36.20%

95% CI, 95% Confidence Interval; OR, Odds Ratio; I^2 =Percentage of the total variability due to between-cohorts heterogeneity; NO_2, nitrogen dioxide; NO_x, nitrogen oxides; p-heter, P value of heterogeneity using the Cochran's Q test; PM_{10}, particle matter less than 10µm; $PM_{2.5}$, particle matter less than 2.5µm; PM_{coarse}, particle matter between 2.5 and 10µm; $PM_{2.5}$absorbance, reflectance of $PM_{2.5}$ filters

[a] Odds Ratio and 95% confidence interval estimated by random-effects meta-analysis by area. Models were adjusted for maternal education, maternal country of birth, maternal age at delivery, maternal pre-pregnancy body mass index, maternal height, maternal smoking during pregnancy, parity, child's sex, urbanicity at child's birth address, child's age at autistic traits assessment, and type of evaluator of the autistic traits test

[b]Number of cohorts included in the meta-analysis

Table S12. Sensitivity analyses of fully adjusted combined associations[a] between air pollution exposure during pregnancy[b] and autistic traits within the borderline/clinical range

Air pollutant	N[c]	OR	(95% CI)	p-heter	I^2
**Among children with stable residence from birth until the autistic traits assessment**					
NO_2 (per Δ10 μg/m^3)	6	0.95	(0.77, 1.17)	0.986	0.00%
NO_X (per Δ20 μg/m^3)	6	1.02	(0.87, 1.20)	0.984	0.00%
PM_{10} (per Δ10 μg/m^3)	4	1.15	(0.68, 1.95)	0.241	28.50%
$PM_{2.5}$ (per Δ5 μg/m^3)	4	1.04	(0.67, 1.62)	0.355	7.54%
PM_{coarse} (per Δ5 μg/m^3)	4	1.21	(0.74, 1.99)	0.166	40.93%
$PM_{2.5}$absorbance (per Δ10^{-5}m^{-1})	4	0.95	(0.66, 1.35)	0.795	0.00%
Traffic intensity on the nearest road (per Δ5000 mv/day)	3	1.03	(0.91, 1.16)	0.583	0.00%
Total traffic load on major road within 100m buffer (per Δ4000000 mv/day*m)	3	0.98	(0.74, 1.31)	0.619	0.00%
**Among children who had mothers with high educational level**					
NO_2 (per Δ10 μg/m^3)	3	0.98	(0.62, 1.56)	0.147	47.79%
NO_X (per Δ20 μg/m^3)	3	1.06	(0.66, 1.71)	0.046	67.48%
PM_{10} (per Δ10 μg/m^3)	3	0.88	(0.37, 2.08)	0.021	73.97%
$PM_{2.5}$ (per Δ5 μg/m^3)	3	0.74	(0.20, 2.72)	0.005	81.13%
PM_{coarse} (per Δ5 μg/m^3)	3	0.98	(0.50, 1.91)	0.080	60.44%
$PM_{2.5}$absorbance (per Δ10^{-5}m^{-1})	3	0.93	(0.34, 2.61)	0.032	70.86%
Traffic intensity on the nearest road (per Δ5000 mv/day)	3	1.02	(0.91, 1.14)	0.398	0.00%
Total traffic load on major road within 100m buffer (per Δ4000000 mv/day*m)	3	1.12	(0.68, 1.84)	0.184	41.00%
**Among children who had mothers who did not smoke during pregnancy**					
NO_2 (per Δ10 μg/m^3)	6	0.94	(0.79, 1.11)	0.589	0.00%
NO_X (per Δ20 μg/m^3)	6	0.97	(0.86, 1.09)	0.654	0.00%
PM_{10} (per Δ10 μg/m^3)	4	0.95	(0.71, 1.28)	0.488	0.00%
$PM_{2.5}$ (per Δ5 μg/m^3)	4	0.86	(0.47, 1.58)	0.099	52.26%
PM_{coarse} (per Δ5 μg/m^3)	4	0.98	(0.71, 1.35)	0.267	23.98%
$PM_{2.5}$absorbance (per Δ10^{-5}m^{-1})	4	0.91	(0.61, 1.36)	0.222	31.78%
Traffic intensity on the nearest road (per Δ5000 mv/day)	2	1.00	(0.90, 1.11)	0.384	0.00%
Total traffic load on major road within 100m buffer (per Δ4000000 mv/day*m)	2	0.96	(0.79, 1.16)	0.329	0.00%

95% CI, 95% Confidence Interval; OR, Odds Ratio; I^2 =Percentage of the total variability due to between-cohorts heterogeneity; NO_2, nitrogen dioxide; NO_x, nitrogen oxides; p-heter, P value of heterogeneity using the Cochran's Q test; PM_{10}, particle matter less than 10μm; $PM_{2.5}$, particle matter less than 2.5μm; PM_{coarse}, particle matter between 2.5 and 10μm; $PM_{2.5}$absorbance, reflectance of $PM_{2.5}$ filters

[a]Odds Ratio and 95% confidence interval estimated by random-effects meta-analysis by area. Models were adjusted for maternal education, maternal country of birth, maternal age at delivery, maternal pre-pregnancy body mass index, maternal height, maternal smoking during pregnancy, parity, child's sex, season at child's birth date, urbanicity at child's birth address, child's age at autistic traits assessment, and type of evaluator of the autistic traits test. Models of traffic indicator variables were additionally adjusted for non-back-extrapolated background levels of NO_2

[b]Air pollution levels were temporally adjusted to the exact pregnancy period except for the traffic indicator variables

[c]Number of cohorts included in the meta-analysis

Table S13. Fully adjusted combined associations[a] between air pollution exposure during pregnancy[b] and autistic traits within the borderline/clinical range by child's sex

	Males					Females				
**Air pollutant**	**N[c]**	**OR**	**(95% CI)**	**p-heter**	**I^2**	**N[c]**	**OR**	**(95% CI)**	**p-heter**	**I^2**
NO_2 (per Δ10 μg/m^3)	6	1.02	(0.82, 1.27)	0.340	11.75%	5	0.85	(0.65, 1.11)	0.567	0.00%
NO_X (per Δ20 μg/m^3)	6	1.10	(0.86, 1.40)	0.115	43.59%	5	0.91	(0.70, 1.10)	0.308	16.68%
PM_{10} (per Δ10 μg/m^3)	4	1.01	(0.66, 1.54)	0.328	12.88%	3	0.82	(0.52, 1.29)	0.683	0.00%
$PM_{2.5}$ (per Δ5 μg/m^3)	4	0.92	(0.42, 2.02)	0.067	58.19%	3	0.72	(0.43, 1.20)	0.361	1.96%
PM_{coarse} (per Δ5 μg/m^3)	4	1.03	(0.70, 1.53)	0.259	25.44%	3	0.93	(0.64, 1.34)	0.865	0.00%
$PM_{2.5}$absorbance (per Δ$10^{-5}m^{-1}$)	4	0.94	(0.46, 1.90)	0.036	64.91%	3	0.65	(0.40, 1.05)	0.944	0.00%
Traffic density on nearest street (per Δ5,000 mv/day)	3	1.05	(0.95, 1.15)	0.870	0.00%	3	0.92	(0.76, 1.11)	0.780	0.00%
Traffic load on all major roads within 100m buffer (per Δ4,000,000 mv/day*m)	3	1.03	(0.65, 1.63)	0.140	49.14%	3	1.07	(0.85, 1.33)	0.460	0.00%

95% CI, 95% Confidence Interval; OR, Odds Ratio; I^2 =Percentage of the total variability due to between-cohorts heterogeneity; NO_2, nitrogen dioxide; NO_x, nitrogen oxides; p-heter, P value of heterogeneity using the Cochran's Q test; PM_{10}, particle matter less than 10μm; $PM_{2.5}$, particle matter less than 2.5μm; PM_{coarse}, particle matter between 2.5 and 10μm; $PM_{2.5}$absorbance, reflectance of $PM_{2.5}$ filters

[a] Odds Ratio and 95% confidence interval estimated by random-effects meta-analysis by area. Models were adjusted for maternal education, maternal country of birth, maternal age at delivery, maternal pre-pregnancy body mass index, maternal height, maternal smoking during pregnancy, parity, child's sex, season at child's birth date, urbanicity at child's birth address, child's age at autistic traits assessment, and type of evaluator of the autistic traits test. Models of traffic indicator variables were additionally adjusted for non-back-extrapolated background levels of NO_2

[b]Air pollution levels were temporally adjusted to the exact pregnancy period except for the traffic indicator variables

[c]Number of cohorts included in the meta-analysis

References

Achenbach TM and Rescorla LA. 2000. Manual for ASEBA preschool forms & profiles. Burlington, VT: University of Vermont, Research Center for Children, Youth, & Families

American Psychiatric Association. 2000. Diagnostic and statistical manual of mental disorders, 4th ed. (DSM-IV). Washington, DC: APA

Anckarsäter H, Lundström S, Kollberg L, Kerekes N, Palm C, Carlström E, et al. 2011. The Child and Adolescent Twin Study in Sweden (CATSS). Twin Res Hum Genet 14(6):495-508

Baron-Cohen S, Scott FJ, Allison C, Williams J, Bolton P, Matthews FE, et al. 2009. Prevalence of autism-spectrum conditions: UK school-based population study. Br J Psychiatry 194(6):500–9

Beelen R, Hoek G, Vienneau D, et al. 2013. Development of NO_2 and NO_x land use regression models for estimating air pollution exposure in 36 study areas in Europe – the ESCAPE project. Atmos Environ 72:10-23

Constantino JN and Gruber, CP. 2005. Social responsiveness scale (SRS); Manual. Los Angeles: Western Psychological Services

Constantino JN and Todd RD. 2000. Genetic structure of reciprocal social behavior. Am J Psychiatry 157:2043–2045

Cyrys J, Eeftens M, Heinrich J, et al. 2013. Variation of NO_2 and NO_x concentrations between and within 36 European study areas: Results from the ESCAPE study. Atmos Environ 63:374-390

Eeftens M, Beelen R, de Hoogh K, Bellander T, Cesaroni G, Cirach M, et al. 2012a. Development of Land Use Regression Models for $PM_{2.5}$, $PM_{2.5}$ Absorbance, PM_{10}

and PM_{coarse} in 20 European Study Areas; Results of the ESCAPE Project. Environ Sci Technol 46:11195-11205

Eeftens M, Tsai MY, Ampe C, et al. 2012b. Spatial variation of $PM_{2.5}$, PM_{10}, $PM_{2.5}$ absorbance and PM_{coarse} concentrations between and within 20 European study areas and the relationship with NO_2 - Results of the ESCAPE project. Atmos Environ 62:303-317

Hansson SL, Svanstrom Rojvall A, Rastam M, Gillberg C, Anckarsater H. 2005. Psychiatric telephone interview with parents for screening of childhood autism-tics, attention-deficit hyperactivity disorder and other comorbidities (A-TAC): preliminary reliability and validity. Br J Psychiatry 187:262-7

Ivanova MY, Achenbach TM, Rescorla LA, Harder VS, Ang RP, Bilenberg N, et al. 2010. Preschool psychopathology reported by parents in 23 societies: testing the seven-syndrome model of the child behavior checklist for ages 1.5–5. J Am Acad Child Adolesc Psychiatry 49:1215–1224.

Larson T, Anckarsater H, Gillberg C, Stahlberg O, Carlstrom E, Kadesjo B, et al. 2010. The autism-tics, AD/HD and other comorbidities inventory (A-TAC): further validation of a telephone interview for epidemiological research. BMC Psychiatry 10:1

Muratori F, Narzisi A, Tancredi R, Cosenza A, Calugi S, Saviozzi I, et al. 2011. The CBCL 1.5–5 and the identification of preschoolers with autism in Italy. Epidemiol Psychiatr Sci 20:329–338

Pedersen M, Giorgis-Allemand L, Bernard C, Aguilera I, Andersen AM, Ballester F, et al. 2013. Ambient air pollution and low birthweight: a European cohort study (ESCAPE). Lancet Respir Med 1:695-704

Román GC, Ghassabian A, Bongers-Schokking JJ, Jaddoe VW, Hofman A, de Rijke YB, et al. 2013. Association of gestational maternal hypothyroxinemia and increased autism risk. Ann Neurol 74(5):733-42

Sikora DM, Hall TA, Hartley SL, Gerrard-Morris AE, Cagle S. 2008. Does parent report of behavior differ across ADOS-G classifications? Analysis of scores from the CBCL and GARS. J Autism Dev Disord 38:440–448

Tick NT, van der Ende J, Koot HM, Verhulst FC. 2007. 14-year changes in emotional and behavioral problems of very young Dutch children. J Am Acad Child Adolesc Psychiatry 46:1333–1340

Williams J, Scott F, Stott C, Allison C, Bolton P, Baron-Cohen S, et al. 2005. The CAST (Childhood Asperger Syndrome Test): test accuracy. Autism 9(1):45-68

World Health Organization. 1993. The ICD-10 Classification of Mental and Behavioural Disorders: Diagnostic Criteria for Research. Geneva, Switzerland: World Health Organization
